# Down-regulation of Fas-L in glioma cells by ribozyme reduces cell apoptosis, tumour-infiltrating cells, and liver damage but accelerates tumour formation in nude mice

**DOI:** 10.1054/bjoc.2001.2055

**Published:** 2001-10

**Authors:** C-C Chio, Y-S Wang, Y-L Chen, S-J Lin, B-C Yang

**Affiliations:** Department of Neurosurgery, Chi-Mei Foundation Hospital; Microbiology and Immunology, Pediatric, College of Medicine, National Cheng Kung University, Tainan, Taiwan, Republic of China

**Keywords:** Fas-L, ribozyme, tumorigenesis, glioma

## Abstract

Fas-L (CD95L, APO-1L) expresses in a variety of tumours and has been proposed to play a role in tumour formation and metastasis. The contribution of Fas-L to tumour growth, however, is not conclusive especially in systems using cells with over-expressed Fas-L. In this study we down-regulated the expression o Fas-L in human glioma cells by a hammerhead ribozyme (Fas-L^ribozyme^) targeting against Fas-L mRNA. Fas-L^ribozyme^-carrying cells exhibited slightly enhanced growth rate and less degree of spontaneous apoptosis in vitro as compared with vector controls. In nude mice, Fas-L^ribozyme^-carrying cells grew faster with lesser apoptosis, formed bigger tumour with significantly fewer infiltrating cells in the tumour area, and triggered relatively milder tumour-associated liver damage than vector controls did. Thus, down-regulation of Fas-L not only improved viability of glioma cells but also reduces local immune responses that may consequently affect tumour formation. Taken together, our findings imply that endogenous expression of Fas-L in malignant cells is not always growth promoting. © 2001 Cancer Research Campaign  http://www.bjcancer.com


*British Journal of Cancer* (2001) **85**(8), 1185–1192
© 2001 Cancer Research Campaign
doi: 10.1054/ bjoc.2001.2055, available online at http://www.idealibrary.com on IDEAL

http://www.bjcancer.com

### Down-regulation of Fas-L in glioma cells by ribozyme reduces cell apoptosis, tumour-infiltrating cells, and liver damage but accelerates tumour formation in nude mice

**C-C Chio[1], Y-S Wang[2], Y-L Chen[2], S-J Lin[3] and B-C Yang[2]**

[1]Department of Neurosurgery, Chi-Mei Foundation Hospital, Departments of [2]Microbiology and Immunology, and [3]Pediatric, College of Medicine, National Cheng Kung University, Tainan, Taiwan, Republic of China

**Summary** Fas-L (CD95L, APO-1L) expresses in a variety of tumours and has been proposed to play a role in tumour formation and metastasis. The contribution of Fas-L to tumour growth, however, is not conclusive especially in systems using cells with over-expressed Fas-L. In this study we down-regulated the expression o Fas-L in human glioma cells by a hammerhead ribozyme (Fas-L$^{ribozyme}$) targeting against Fas-L mRNA. Fas-L$^{ribozyme}$-carrying cells exhibited slightly enhanced growth rate and less degree of spontaneous apoptosis in vitro as compared with vector controls. In nude mice, Fas-L$^{ribozyme}$-carrying cells grew faster with lesser apoptosis, formed bigger tumour with significantly fewer infiltrating cells in the tumour area, and triggered relatively milder tumour-associated liver damage than vector controls did. Thus, down-regulation of Fas-L not only improved viability of glioma cells but also reduces local immune responses that may consequently affect tumour formation. Taken together, our findings imply that endogenous expression of Fas-L in malignant cells is not always growth promoting. © 2001 Cancer Research Campaign http://www.bjcancer.com

**Keywords**: Fas-L; ribozyme; tumorigenesis; glioma

Fas/Fas-L signalling system plays an important role in immune homeostasis (Nagata, 1997). Fas-L (also termed as CD95L, APO-1L) is a member of the tumour necrosis factor receptor super-family and may trigger a death signal into Fas-bearing cells after engagement with Fas molecule (Itoh et al, 1991; Takahashi et al, 1994). Contrast to a well-regulated manner in lymphocytes (Alderson et al, 1995; Van Parijs and Abbas, 1996), Fas-L is constitutively expressed in several non-lymphoid tissues as well as in tumours of various origins. The expression of functional Fas-L on some tumour cells may confer them the ability to kill T cells in vitro (Hahne et al, 1996; Strand et al, 1996; Walker et al, 1998). These findings led to an assumption that Fas-L might contribute to immune privilege of tumours. However, using Fas-L-positive cells for organ transplantation or ectopic expression of Fas-L in transgenic animals revealed that Fas-L could also enhance immune destruction of Fas-L-positive cells (Chervonsky et al, 1997; Seino et al, 1997). Under certain growth conditions, cross-linking of Fas transmits signal for proliferation instead of death signal in tumours as well as in immune cells (Alderson et al, 1993, Owen-Schaub et al, 1994). More complicatedly, a reverse signalling of Fas-L has been reported in cytotoxic lymphocytes, where Fas-L augmented cell proliferation following its ligation (Suzuki and Fink, 1998).

Glioma cells express Fas-L and induce apoptosis in Fas-expressing cell lines ex vivo (Gratas et al, 1997; Saas et al, 1997; Yang et al, 2000). While, glioma cells can also express Fas and are susceptible to Fas-L-triggered death indicating that a subtle balance of growth and death signals exists in vivo (Weller et al, 1994; Yang et al, 1999). Conversely, endogenous Fas/Fas-L interaction in glioma cells could provide signal driving cell cycle progression in glioma cells probably through the MEK-ERK pathway (Shinohara et al, 2000). In this study, we constructed a Fas-L-specific hammerhead ribozyme (Fas-L$^{ribozyme}$), placed upstream of enhanced green fluorescent protein (EGFP) gene to suppress the Fas-L expression in human glioma cells. Ribozyme catalyses endoribonucleolytic cleavage of substrate RNA in trans with a sequence-specific manner (Birikh et al, 1997; Poeschla and Wong-Staal, 1997) and has been exploited to inhibit genes such as *fos, ras* and *fas-L* (Scanlon et al, 1991; Du et al, 1996; Chang et al, 1997). Our Fas-L$^{ribozyme}$ recognizes the Fas-L transcript at site coding for the extracellular domain of Fas-L. After transfection into glioma cells, Fas-L$^{ribozyme}$ inhibited the expression of Fas-L at both mRNA and protein levels. Stable transfectants of glioma cells carrying Fas-L$^{ribozyme}$ were established to explore the role of Fas-L on cell growth, apoptosis, and tumour formation in nude mice. Here, we present evidence that down-regulation of endogenous Fas-L in tumour cells not only affects viability of tumour cells but also reduces local immune responses that may consequently modify tumour formation.

### MATERIALS AND METHODS

### Cells and cell culture condition

Human glioma cell lines U-373MG and U-118MG were purchased from the American Type Culture Collection (Rockville, MD). Transfectants carrying plasmid pEGFP-N1 (vector controls) or

*Received 7 September 2000*
*Revised 10 May 2001*
*Accepted 30 May 2001*

*Correspondence to:* B-C Yang

Fas-L$^{ribozyme}$ were established in this study. Cells were cultured in Dulbecco modified Eagles' medium (DMEM; Life Technologies, Grand Island, NY) supplemented with 20% fetal calf serum (FCS), 1% penicillin and 1% fungizone at 5%$CO_2$ /37°C in a humidified atmosphere. Growth rate was measured by counting viable cell, which was determined by trypan blue exclusion. MTT assay was applied to estimate cellular viability using a commercially available kit (CellTiter 96® Aqueous, Promega, Madison, WI). In brief, cells were seeded in 96-well plates ($5 \times 10^4$ cells well$^{-1}$), grew for 72 h to confluence, and the WST-8 (2-(2-methoxy-4-nitrophenyl)-3-(4-nitrophenyl)-5-(2,4-disulfophenyl)-2H-tetrazolumn) was added the last 2 h before the end of culture. The cell-bound dye was measured by optical absorption at 490 nm using a microplate reader.

### Construction of Fas-L$^{ribozyme}$

The sequences of the oligonucleotides used to construct Fas-L$^{ribozyme}$ were as follow: sense sequence: 5′-ATG/AAT/TCC/CGG/AAG/TAC/TGA/TGA/GTC/GTC/ATA/CGA/ CGA/AAC/TTT/GGA/ TCC/CGA-3′; antisense sequence: 5′-TCG/GGA/TCC/AAA/GTT/TCG/CG/TAT/CAC/GAC/TCA/TCA/GTA/CTT/CCG/GGA/ATT /CAT-3′. This sequence is corresponding to the coding region in the extracellular domain of Fas-L and does not share homology to other genes according to database search on GenBank, EMBL, PDB and DDBJ. After annealing of these oligonucleotide pairs, the generated fragment was digested with *EcoR1* and *BamH1*, followed by ligation into the *EcoR1/BamH1*-predigested vector plasmid pEGFP-N1 (Clontech, palo Atto, CA). In this plasmid construct, the ribozyme was directly linked to upstream of the *EGFP* gene to form a fusion transcript (Figure 1), so that the Fas-L$^{ribozyme}$ can be monitored indirectly by the expression of EGFP protein. Candidate clone was verified by DNA sequencing.

### DNA transfection

Plasmid DNA was delivered into cells using the lipofection method with a ratio of 1 µg DNA /20 µl lipofectamine (Qiagen, Hilden, Germany). Plasmid pEGFP-N1 served as the non-ribozyme control. After DNA transfection, cells were grown in regular 20% FCS/DMEM for 48 h, and then selected with geneticin (G418, Sigma, St Louis, MO) at an effective concentration of 0.5 µg ml$^{-1}$ for at least 3 months before subjected to further study. Stable transfectants derived from U-373MG and U 118MG carrying either Fas-L$^{ribozyme}$ or pEGFP-N1 plasmid were designated as Fas-L$^{ribozyme}$ (U-373MG), Fas-L$^{ribozyme}$ (U-118MG), EGFP(U-373MG) and EGFP(U-118MG), respectively.

### Semi-quantitative reversed transcription-polymerase chain reaction (RT-PCR)

Total RNA was purified using the RNeasy Kit followed the manufacturer's instruction (Qiagen) and converted to cDNA by StrataScrip™-H-reverse transcriptase with oligo-dT primer in the presence of RNAsin (Stratagen, La Jolla, CA). RT-PCR for Fas-L, Fas, Bcl-2, FAP-1 and β-actin were performed as described previously (Cleary et al, 1986; Sato et al; 1995; Yang et al, 1998). The generated cDNA was subjected to 35–40 cycles of PCR amplification on a DNA thermal Cycler (Hybaid Omnigene, Middlesex, UK). The existence of ribozyme in stable cells was verified by

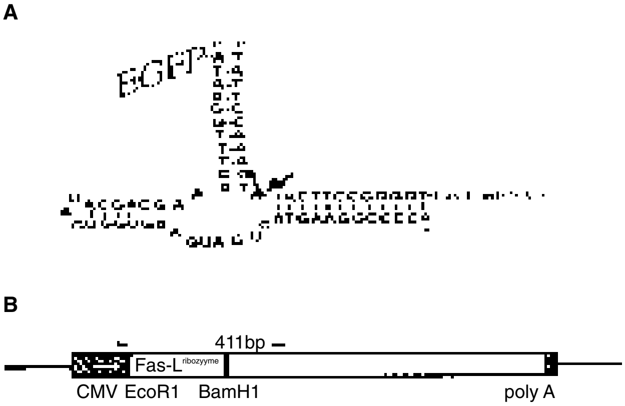

**Figure 1** Construction of Fas-L$^{ribozyme}$. (**A**) Secondary structure of ribozyme that cleaves the Fas-L mRNA at the coding region in the extracellular domain of Fas-L. Arrow indicates the cleavage site. This Fas-L$^{ribozyme}$ is fused upstream with *EGFP* gene. (**B**) Plasmid EGFP/Fas-L$^{ribozyme}$ was constructed by ligation of the oligos encoding ribozyme sequence into EcoR1, BamH1-digested pEGFP-N1. Arrows indicate the positions of primer set detecting the chimaeric transcript (Fas-L$^{ribozyme}$/EGFP) in PCR reactions

RT-PCR with primer pair as follow: 5′-TCC/GCT/AGC/GCT/ACC/GGA/CT- 3′ and 5′-GTC/CTT/GAA /GAA/GAT/GGT-3′. This primer pair amplifies a 411 bp from the EGFP/Fas-L$^{ribozyme}$ chimeric transcript spanning upstream of ribozyme into EGFP gene, and a 400 bp DNA fragment from the transcript of authentic *EGFP* gene. β-Actin served as a quantitative control in PCR. PCR products were fractionated by agarose or NuSieve 3:1 agarose, stained with ethidium bromide, and visualized under UV light.

### Western blot

Cells were extracted with a buffer containing 20 mM Tris, 150 mM NaCl, 1 mM EDTA, 1% Nonidet P-40, 1 mM PMSF and 0.1 U ml$^{-1}$ leupeptin. Proteins were separated in SDS-polyacrylamide gel and electroblotted onto polyvinyl difluoride membrane (MSI, Westboro, MO). The proteins bounded on the membrane were probed with mouse antibody (Ab)-recognizing Fas-L (clone33; Transduction Laboratories, Lexington, KY) followed by a sheep anti-mouse IgG conjugated with horseradish peroxidase (Dako Corp, Carpinteria, CA). The Fas-L was made visible by fluorography with enhanced chemiluminescence detection kit (Amersham/Pharmacia Biotech, UK). Duplicate blot was probed with α-tubulin-specific Ab (clone DMIA; NeoMarker, Fremont, CA) and served as a protein-loading control.

### Detection of apoptotic cells

Apoptotic cells in 3-day-old culture were stained with merocyanine 540 (MC540, Sigma) according to the published procedures (Reid et al, 1996; Yang et al, 1999). Apoptotic cells became susceptible to MC540 binding due to alteration of surface membrane and showed FL2 values more than 230 detected by flow cytometry analysis. Apoptotic cells in tumour nodules or liver tissues were detected by TUNEL labelling detecting free 3′-OH groups in fragmented DNA in situ (Apop Tag-peroxidase in situ apoptosis detection kit, Oncor, MD). Paraffin-embedded, slide-mounted tissue sections were deparaffinized, and treated with proteinase K for 15 min followed by 3% $H_2O_2$ for 5 min. After nick end labelling with digoxigenin-deoxyuridine triphosphate by deoxynucleotidyl transferase, immunostaining was

 © 2001 Cancer Research Campaign performed using peroxidase-conjugated antidigoxigenin Ab for 30 min. Apoptotic cells were visualized with diaminobenzidine substrate and became a dark brown colour. Specimen was counter-stained with 0.3% methyl green or haematoxylin/eosin.

### Tumour formation in nude mice

Male BALB/c-nu mice were purchased from the National Laboratory Animal Breeding and Research Center, Taiwan, ROC and maintained under specific pathogen-free condition. Mice at 8 weeks old were randomly divided into groups and 3–5 mice in a group. Approximate $10^6$ stable cells in 0.3 ml PBS were injected subcutaneously into the upper flanks of each mouse. Tumour formation was examined every other day. Injected cells were considered as tumorigenic if tumour nodule with a size more than 3 mm developed at the injection site. Mice were sacrificed one week after tumour nodules were formed. Tumour nodules and liver tissue were surgically obtained, fixed in neutral-buffered formalin, pH 7.2, and then subjected to analyses on tumour-infiltrating cells and apoptotic cells. Infiltrating macrophages and granulocytes were morphologically identified based on the ratio of nuclear to cyto-plasm and the presence or absence of granules. EGFP-expressing cells in those tissues were immunostained using rabbit anti-EGFP Ab (Clontech) followed by peroxidase-conjugated goat anti-rabbit IgG Ab (Calbiochem, La Jolla, CA). Sections were counterstained and mounted with glycerol gelatin before examination.

### Statistical analysis

Results were analysed by Student's *t*-test. Differences with $P < 0.05$ were judged significant.

### RESULTS

### Fas-L$^{ribozyme}$ plasmid reduced the expression of Fas-L in glioma cells

After growing in geneticin-containing medium for 3 months, more than 90% of stable transfectants carrying either Fas-L$^{ribozyme}$ or pEGFP-N1 plasmid emitted green light under fluorescent microscopy and were morphologically similar to the parental cells. Bulk culture of DNA-transfected cells was used in this study to avoid any bias deduced from cell lines having unwanted gene backgrounds generated during selection. The chimeric transcripts containing Fas-L-specific ribozyme and EGFP sequence were detected in Fas-L$^{ribozyme}$-carrying cells by RT-PCR as a 411 bp DNA fragment (Figure 2A). A 400 bp PCR fragment was ampli-fied from the cDNA of vector controls, which represents the authentic transcript of the *EGFP* gene. Transfection of Fas-L$^{ribozyme}$ did not affect the amount of Fas, Bcl-2 and FAP-1 transcripts in both U-118MG and U-373MG cells (Figure 2B). A decrease in the expression of Fas-L was observed in Fas-L$^{ribozyme}$-carrying cells as compared to those in vector controls at both mRNA (Figure 2B) and protein (Figure 2C) levels.

### Fas-L$^{ribozyme}$ enhanced slightly the growth rate but reduced spontaneous apoptosis of glioma cells in vitro

Growth rate of stable transfectants was determined at 24, 48 and 72 h after subculture in fresh medium (Figure 3). Fas-L$^{ribozyme}$(U-373MG) showed enhanced growth rate in vitro than EGFP(U-373MG). In

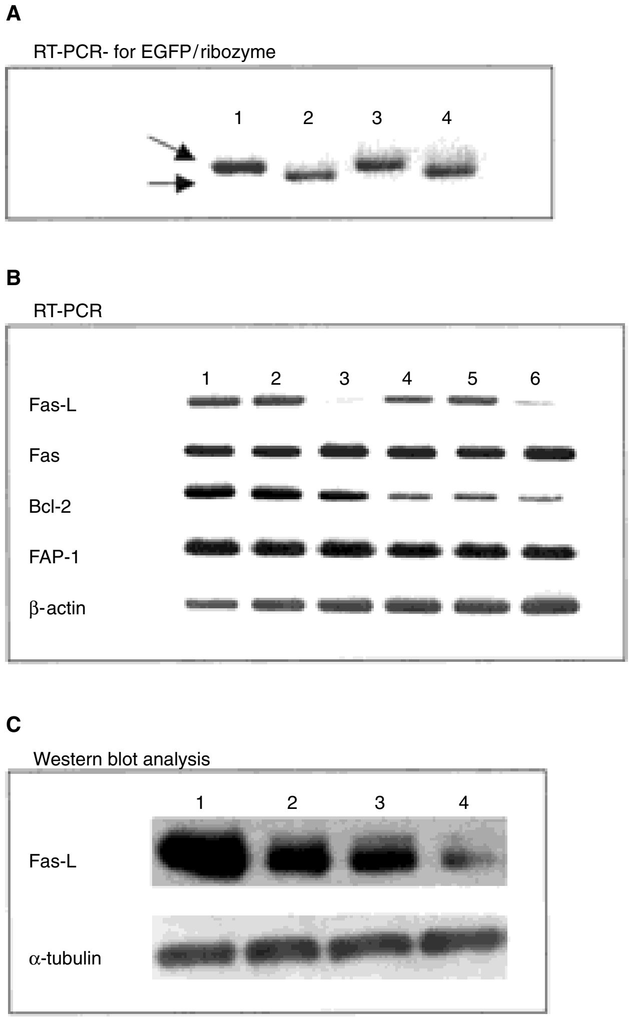

**Figure 2** Decreased expression of Fas-L in Fas-$^{ribozyme}$-carrying cells. After transfection, cells were selected in geneticin-containing medium for 3 months. RNA was isolated and converted to cDNA as described in Materials and Methods. (**A**) PCR-amplified products of chimeric Fas-L$^{ribozyme}$ (411 bp) in Fas-L$^{ribozyme}$(U-373MG) and Fas-L$^{ribozyme}$(U-118MG) (lanes 1, 3), or EGFP (400 bp) in EGFP(U-373MG) and EGFP(U-118MG) (lanes 2, 4). (**B**) PCR-amplified products of Fas-L, Fas, Bcl-2, FAP-1 and β-actin. Lanes 1, 2, 3: U-118MG, EGFP(U-118MG) and Fas-L$^{ribozyme}$(U-118MG), respectively; lanes 4, 5, 6: U-373MG, EGFP(U-373MG) and Fas-L$^{ribozyme}$(U-373MG), respectively. (**C**) Western blot analysis. Lanes 1, 2: EGFP(U-373MG), Fas-L$^{ribozyme}$(U-373MG); lanes 3, 4: EGFP(U-118MG), Fas-L$^{ribozyme}$(U-118MG), respectively. α-Tubulin served as protein-loading control general, Fas-L$^{ribozyme}$-carrying cells grew slightly faster than vector controls, although elevation in growth rate in Fas-L$^{ribozyme}$(U-118MG) was not statistically significant. In addition, less floated cells and apoptotic bodies appeared in 72-h culture of Fas-L$^{ribozyme}$-glioma cells than that of vector controls. The viability of the trans-fectants in 72-h culture, in which cells grew to confluence, was estimated by MTT assay (Figure 4). Fas-L$^{ribozyme}$(U-373MG) showed significantly better viability than EGFP(U-373MG). In consistent with the growth rate, cellular viability of Fas-L$^{ribozyme}$(U-118MG) was not significant different from that of EGFP(U-118MG). To further quantify the apoptosis, cells were stained with (MC540) (Figure 5). The proportion of MC540-positive cells were 8.7% in culture of Fas-L$^{ribozyme}$(U-373MG) as compared to 38.5% in culture of EGFP(U-373MG). Similarly, MC540-positive cells in Fas-L$^{ribozyme}$(U-118MG) were about 3-fold less than

© 2001 Cancer Research Campaign

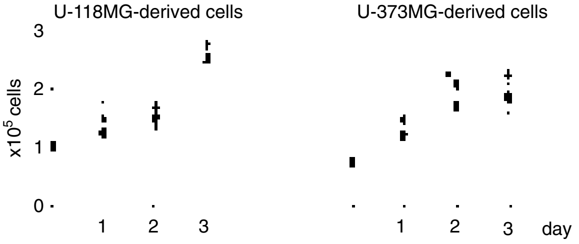

**Figure 3** Growth curves of cells with or without Fas-L$^{ribozyme}$. Cells were grown in 20% FCS/DMEM at the initial density of $1 \times 10^5$ cells well$^{-1}$ in a 6-well plate. Cell number was counted every 24 h up to 72 h. Diamonds: cells carrying Fas-L$^{ribozyme}$; Squares: cells carrying EGFP-N1 plasmid. $^*$:significant difference among groups ($P < 0.05$)

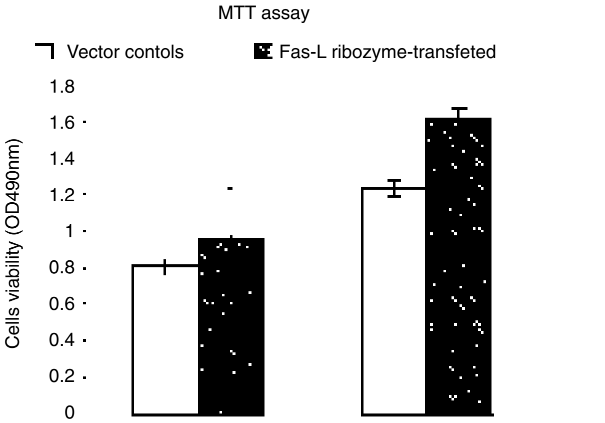

**Figure 4** Cellular viability. About $5 \times 10^4$ cells were grown in 20% FCS/DMEM for 72 h in a well of a 96-well plate. Cellular viability was determined by MTT assay as described in Materials and Methods. Blank bar: cells carrying EGFP-N1 plasmid served as controls; hatched bars: cells carrying Fas-L$^{ribozyme}$. Bars are means ± SD from 3 independent experiments

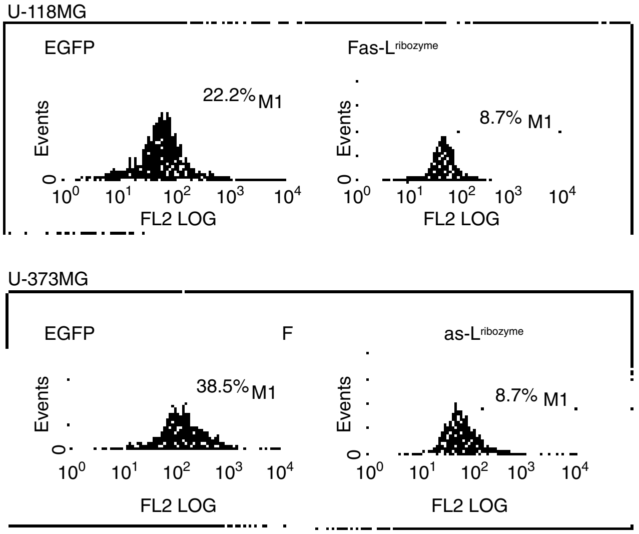

**Figure 5** Apoptosis in 3-day culture of cells with or without Fas-L$^{ribozyme}$. Cells, with (solid lines) or without (filled gray curves) Fas-L$^{ribozyme}$, were grown in 20% FCS/DMEM for 72 h. Apoptotic cells were stained by MC540. Cells with FL2>230 were judged as apoptotic

EGFP(U-118MG) (8.7% versus 22.2%). These results indicate that as a result of Fas-L down-regulation, the Fas-L$^{ribozyme}$ transfectants were more resistant to spontaneous cell death under confluent growth condition.

### Tumour formation of EGFP/Fas-L$^{ribozyme}$ stable cells

U-118MG produced tumours by 2–3 weeks after subcutaneous inoculation into nude mice. Parental U-373MG cells were less tumorigenic and produced tumours in mice around 8–9 weeks after cell injection. To determine whether endogenous tumour Fas-L involves in tumorigenesis, $10^6$ cells, with or without Fas-L$^{ribozyme}$, were injected subcutaneously into the dorsal flanks of nude mice. Results are summarized in Table 1. Fas-L$^{ribozyme}$(U-118MG) and EGFP(U-118MG) produced tumours in all animals by 2 weeks post-injection with diameters of 3–5 mm and 2–4 mm, respectively. EGFP(U-373MG), as expected, produced tumours of small size (1.5–2 mm) and only 2 out of 3 mice developed tumours first at 7.5 and 10 weeks post-injection. Interestingly, Fas-L$^{ribozyme}$(U-373MG) produced more tumours in all injected mice with diameters of 3–4 mm about 3–5 weeks earlier.

### Histopathology

Fas-L$^{ribozyme}$ altered the architecture of tumours. Tumour nodules of Fas-L$^{ribozyme}$-carrying cells showed loose margin and invasive characters (Figure 6). Immune cells showing morphology of mononuclear cells/granulocytes accumulated in and around the tumour nodules (Figure 7). There were less infiltrating cells in tumours produced by Fas-L$^{ribozyme}$ carrying cells than those of vector controls (Table 2). Notably, the infiltrating cells tended to accumulate near the margin of tumour nodules produced by vector controls (Figure 7B). Apoptotic cells showing fragmented nuclei and stained positive by TUNEL staining (brown-coloured cells as shown in the representative pictures for U-373MG; Figure 8) were numerous in tumours of EGFP(U-118MG) and EGFP(U-373MG). Apoptosis was significantly reduced in tumour nodules of all Fas-L$^{ribozyme}$-carrying cells as compared to vector controls.

### Liver damage in tumour-bearing mice

Multifocal, diffuse, necrotizing areas were frequently observed in liver of mice bearing vector controls (Figure 9A, C), but less in liver of mice bearing Fas-L$^{ribozyme}$-carrying cells (Figure 9B). Apoptotic cells showing morphological features of hepatocytes were demonstrable in the liver lesion (Figure 9D). Take the advantage that our established tumour cell lines express EGFP, the presence of tumour cells in situ was confirmed by histological

**Table 1** Summary of the effects of Fas-L$^{ribozyme}$ on tumour formation

| | U-373MG | | U-118MG | |
|---|---|---|---|---|
| | Control | Fas-L$^{ribozyme}$ | Control | Fas-L$^{ribozyme}$ |
| Tumour size ($mm^3$) | 1.5–2 | 3–4 | 2–4 | 3–5 |
| Tumour number (total in 3 mice) | 2 | 7 | 5 | 5 |
| Tumour formation (weeks) | 7.5–10 | 4.5–5 | 2 | 2 |
| Infiltrating cells | +++ | + | +++ | + |
| Apoptotic cells | +++ | ++ | +++ | + |
| Liver damage | ++ | + | +++ | + |

Note: +++: severe; ++: moderate; +: mild; –/+: few; –: not found.

*© 2001 Cancer Research Campaign*

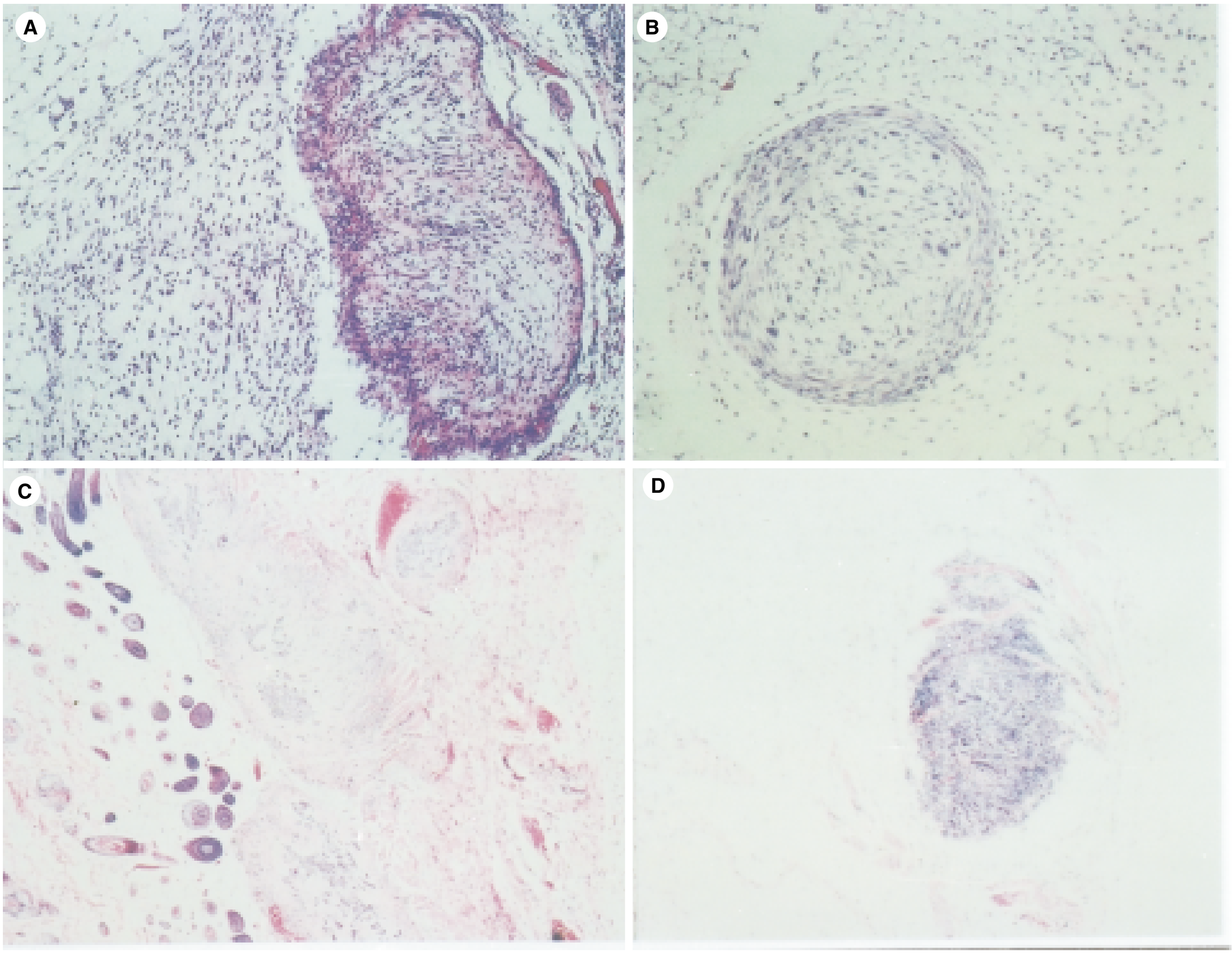

**Figure 6** Tumour formation of U-118MG- and U-373MG-derived cells in nude mice. Approximate $10^6$ stable cells, EGFP(A:U-118MG, C:U-373MG) or Fas-L$^{ribozyme}$(B:U-118MG, D:U-373MG), were injected subcutaneously into dorsal flanks of nude mice. Tumour tissues were formalin-fixed, paraffin-embedded, and stained with haematoxylin/eosin

**Table 2** Tumour-infiltrating cells

| | U-118MG | | U-373MG | |
|---|---|---|---|---|
| | **Ribozyme** | **Vector** | **Ribozyme** | **Vector** |
| 1/T ratio[a] | 0.085 ± 0.059 | 0.162 ± 0.051* | 0.041 ± 0.023 | 0.140 ± 0.058* |
| G/T ratio | 0.008 ± 0.004 | 0.053 ± 0.017* | 0.002 ± 0.004 | 0.053 ± 0.036* |
| M/T ration | 0.037 ± 0.012 | 0.091 ± 0.049* | 0.037 ± 0.021 | 0.082 ± 0.033* |

[a]I/T: infiltrating cells/total cells; G/T: granulocytes/total cells; M/T: macrophages/total cells. Each value represents mean ± SD obtained from 3 mice of all groups except U-373MG/vector, the latter were obtained from 2 tumour-bearing mice. Infiltrating cells were randomly counted in 20 fields for each mouse. *: Significant difference between ribozyme-carrying glioma cells and control groups ($P < 0.05$).

immunostaining on EGFP protein (Figure 9E, F). Although all EGFP-positive glioma cells, both Fas-L$^{ribozyme}$-carrying cells and vector controls, did not form gross detectable tumour nodules in the liver by subcutaneous injection, a few EGFP-positive tumour cells were found among the liver lesions and were morphologically different from those in subcutaneous tumour nodules (comparing Figure 9E).

### DISCUSSION

By using a hammerhead ribozyme we have successfully down-regulated the expression of Fas-L in human glioma cells at both mRNA and protein levels. Apoptosis in tumour cells was significantly reduced by Fas-L$^{ribozyme}$. In nude mice, Fas-L$^{ribozyme}$-carrying cells formed bigger tumours with significantly fewer infiltrating cells in the tumour areas than vector controls.

Fas/Fas-L signalling has been demonstrated to elicit apoptosis in glioma cells in certain culture conditions (Weller et al, 1994; Shinoura et al, 1998; Yang et al, 1999). Although cell cycle progression in glioma cells could be promoted by activating Fas antibody (CH-11) probably through the MEK-ERK pathway, however, apoptosis occurred at the same time and the net viable cell count was not increased (Shinohara et al, 2000). In addition, transfer of *Fas-L* gene into Renca cells, a Fas signal-sensitive renal cell carcinoma caused a suicidal destruction of tumour cells by apoptosis (Arai et al, 1997). In consistence with those findings, glioma cells carrying Fas-L$^{ribozyme}$ had reduced apoptosis both in vitro and in vivo. Since Fas-L$^{ribozyme}$ did not alter the expression of other death-related genes including Fas, Bcl-2, and FAP-1, suppression of *Fas-L* gene by Fas-L$^{ribozyme}$ resulting in less Fas/Fas-L ligation should account for the reduced apoptosis in

© 2001 Cancer Research Campaign

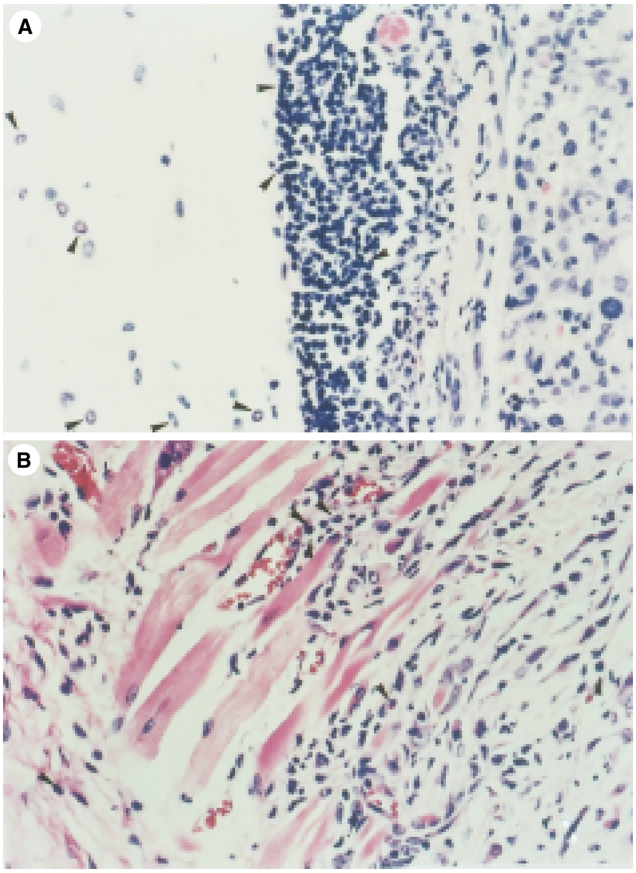

**Figure 7** Tumour-infiltrating cells. Tumour formation and cell staining were done with same procedures as described in Figure 5. Arrowheads indicate tumour-infiltrating cells. (**A**) EGFP(U-373MG); (**B**) Fas-L$^{ribozyme}$(U-373MG); 400x

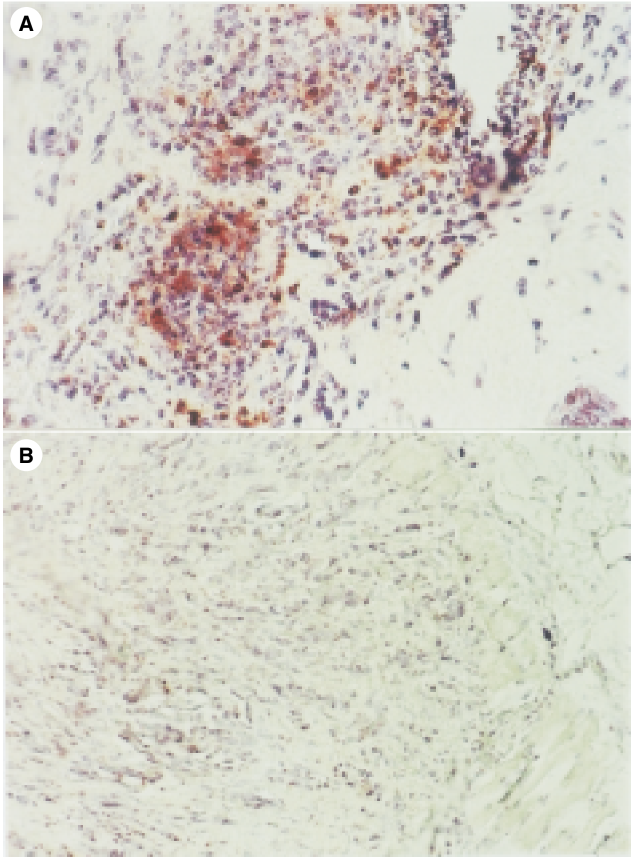

**Figure 8** Apoptotic cells in tumour nodules. Apoptotic cells were detected by Apop Tag-peroxidase in situ apoptosis detection kit. DNA strand break in apoptotic cells was labelling and made visible as described in Material and Methods. Apoptotic cells revealed brown colour. (**A**) EGFP(U-373MG); (**B**) Fas-L$^{ribozyme}$(U-373MG); 400x those Fas-L$^{ribozyme}$-carrying glioma cells. Down-regulation of Fas-L by Fas-L$^{ribozyme}$ caused an increase in growth rate, improved cell viability and speeded up tumour formation indicating that the endogenous tumour Fas-L might not be growth promoting for glioma cells in all. Recently, we had observed that delivering Fas-L$^{ribozyme}$ into murine melanoma or Ras-activated NIH3T3 malignant cell lines also effectively reduced apoptosis in those cells (our unpublished data). It seems that the suicidal effect triggered by Fas-L occur widely in many malignant cells.

The proinflammatory effect of Fas-L has been recognized for days and is attributed partly to local recruitment and activation of neutrophils (Seino et al, 1997; Chen et al, 1998). Apoptotic body by itself is a potent chemotactic agent for immune cells (Horino et al, 1998). Besides, when phagocytes engulf apoptotic body, they can effectively initiate T-cell immunity (Chattergoon et al, 2000). Therefore, the Fas-L$^{ribozyme}$-associated suppression in tumour-infiltrating cells in nude mice observed in our system could be due to reduced amount of apoptotic body generated by Fas-L$^{ribozyme}$-carrying cells. Alternatively, Fas/Fas-L engagement between tumour and immune cells in subcutaneous environments will trigger survival favouring signal for cells constituting innate immunity as those cases reported for T cells or tumours (Alderson et al, 1993, Owen-Shaub et al, 1994). This possibility waits to be proven by additional study. It is noteworthy that U-373MG, U-118MG and their derivatives tested did not form large tumours in nude mice. It substantiates the effectiveness of the remaining innate immunity in nude mice for tumour control and partly explains the negative correlation between cell infiltration and tumour size in this study. Taken together, Fas-L$^{ribozyme}$ could modify the tumorigenesis of human glioma cells in nude mice by several ways. First, Fas-L$^{ribozyme}$ accelerated tumour cell growth by improving cell viability. Second, Fas-L$^{ribozyme}$ reduced the innate immune reaction against tumour.

The appearance of glioma cells in the liver of tumour-bearing mice was an unexpected finding. Even though tumour nodules were not detected grossly in the liver, EGFP-expressing tumour cells were detectable in this tissue. The glioma cells in liver lesion were with atypical shape, mixed with apoptotic hepatocytes and numerous inflammatory cells indicating rigorous local immune responses. Growth of Fas-L$^{+}$ tumour cells in syngeneic murine host has been shown to induce toxicity in Fas$^{+}$ organs probably through the production of soluble Fas-L (Zeytum et al, 2000). Hepatocytes are known to express Fas and are very sensitive to Fas-L-mediated cytotoxicity (Ogasawara et al, 1993; Rensing-Ehl et al, 1995). The expression of Fas-L in human colon cancer has been correlated with the hepatic metastasis and the apoptosis of hepatocytes in tumour lesion (Shiraki et al, 1997). In our study model, Fas-L$^{ribozyme}$ reduced the severity of liver damage in tumour-bearing mice. Therefore, the killing of hepatocytes in glioma-bearing mice was obviously mediated by a contact with

 *© 2001 Cancer Research Campaign*

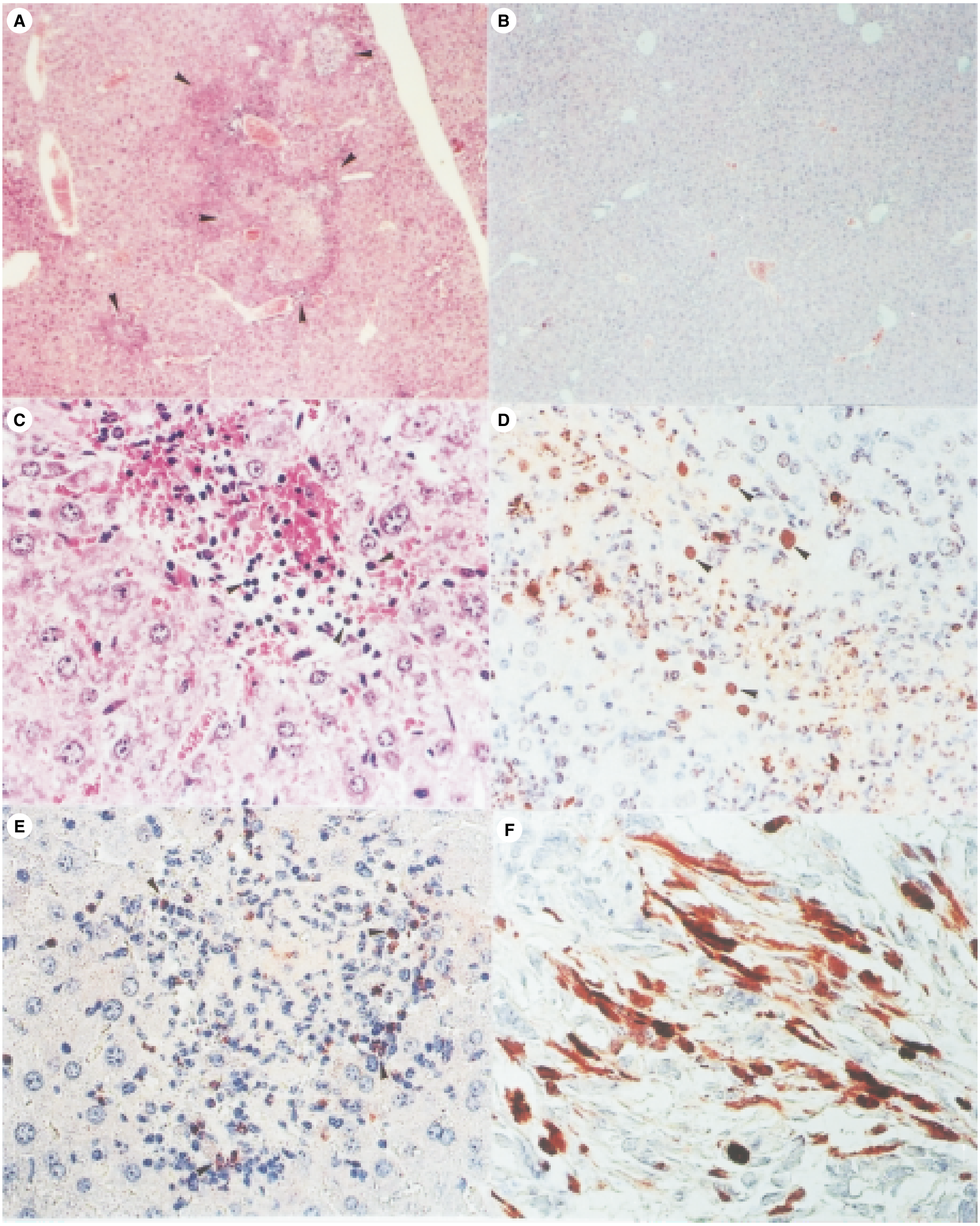

**Figure 9** Liver lesions in mice bearing U-373MG-derived cells. Formalin-fixed, paraffin-embedded liver tissues were prepared from tumour-bearing mice. (**A**, **B**) haematoxylin/eosin-staining (40x). (**A**) EGFP(U-373MG); (**B**) Fas-L$^{ribozyme}$(U-373MG). Arrowheads indicate some damaged tissue regions. (**C**) haematoxylin/eosin-staining of necrotizing area (400x), arrowheads marked some infiltrating immune cells. (**D**) Apoptotic cells were detected by ApopTag-peroxidase in situ apoptosis detection kit. Apoptotic hepatocytes (400x) are indicated by arrowheads. (**E**) EGFP-expressing cells (brown colour) in liver lesion (400x).(**F**) EGFP-expressing cells (brown colour) in tumour nodule (400x)

either metastatic tumour cells or the infiltrating immune cells. Overall, this work using a loss-of-function model appears to complement previous Fas-L overexpression study and implies a possible immune-stimulating function of Fas-L.

### ACKNOWLEDGEMENTS

This work was supported by grants from the National Science Council, ROC, to BC Yang (NSC 88-2316-B006-MB007) and the

© 2001 Cancer Research Campaign 

Chi-Mei Foundation Hospital to CC Chio/BC Yang. We are grateful to Drs JC Yu and CC Shieh for critical reading of the manuscript and Miss YF Lu and WY Shieh for technical assistance.

### REFERENCES

Alderson MR, Armitage RJ, Maraskovsky EW, Tough T, Roux E, Schooley K, Ramsdell F and Lynch DH (1993) Fas transduces activation signals in normal human T lymphocytes. *J Exp Med* **178**: 2231–2235

Alderson MR, Tough TW, Davis-Smith T, Braddy S, Falk B, Schooley KA, Goodwin RG, Smith CA, Ramsdell F and Lynch DH (1995) Fas ligand mediates activation-induced cell death in human T lymphocytes. *J Exp Med* **181**: 71–77

Arai H, Gordon D, Nabel EG and Nabel GJ (1997) Gene transfer of Fas ligand induces tumor regression in vivo. *Proc Natl Acad Sci USA* **94**: 13862–13867

Birikh KR, Paul A and Eckstein HF (1997) The structure, function and application of the hammerhead ribozyme. *Eur J Biochem* **245**: 1–16

Chang MY, Won SJ and Liu HS (1997) A ribozyme specifically suppresses transformation and tumorigenicity of Ha-ras oncogene transformed NIH-3T3 cell lines. *J Cancer Res Clin Onc* **123**: 91–99

Chattergoon MA, Kim JJ, Yang JS, Robinson TM, Lee DJ, Dentchev T, Wilson DM, Ayyavoo V and Weiner DB (2000) Targeted antigen delivery to antigen-presenting cells including dendritic cells by engineered Fas-mediated apoptosis. *Nature Biotech* **18**: 974–979

Chen JJ, Sun Y and Nabel GJ (1998) Regulation of the proinflammatory effects of Fas ligand (CD95L). *Science* **282**: 1714–1717

Chervonsky AV, Wang Y, Wong FS, Visintin I, Flavell RA, Janeway CA and Matis LA (1997) The role of Fas in autoimmune diabetes. *Cell* **89**: 17–24

Cleary ML, Smith SD and Sklar J (1986) Cloning and structural analysis of cDNAs for bcl-2 and a hybrid bcl-2/immunoglobulin transcript resulting from the t(14;18) translocation. *Cell* **47**: 19–28

Du Z, Ricordi C, Podack E and Pastori RL (1996) A hammerhead ribozyme that cleaves perforin and fas-ligand RNAs in vitro. *Biochem Biophy Res Comm* **226**: 595–600

Gratas C, Tohma Y, Van Meir EG, Klein M, Tenan M, Ishii N, Tachibana O, Kleihues P and Ohgaki H (1997) Fas ligand expression in glioblastoma cell lines and primary astrocytic brain tumors. *Brain Pathol* **7**: 863–869

Hahne M, Rimoldi D, Schroter M, Romero P, Schreier M, French LE, Schneider P, Bornand T, Fontana A, Lienard D, Cerottini J and Tschopp J (1996) Melanoma cell expression of Fas (Apo-I/CD95) ligand: implications for tumor immune escape. *Science* **274**: 1363–1366

Horino K, Nishiura H, Ohsako T, Shibuya Y, Hiraoka T, Kitamura N and Yamamoto T (1998) A monocyte chemotactic factor, S19 ribosomal protein dimer, in phagocytic clearance of apoptotic cells. *Lab Invest* **78**: 603–617

Itoh N, Yonehara S, Ishii A, Yonehara M, Mizushima S, Sameshima M, Hase A, Seto Y and Nagata S (1991) The polypeptide encoded by the cDNA for human cell surface antigen Fas can mediate apoptosis. *Cell* **66**: 233–243

Nagata S (1997) Apoptosis by death factor. *Cell* **88**: 355–365

Ogasawara J, Watanabe-Fukunaga R, Adachi M, Matsuzawa A, Kasugai T, Kitamura Y, Itoh N, Suda T and Nagata S (1993) Lethal effect of the anti-Fas antibody in mice. *Nature* **364**: 806–809

Owen-Schaub LB, Radinsky R, Kruzel E, Berry K and Yonehara S (1994) Anti-Fas on nonhematopoietic tumors: levels of Fas/APO-1 and bcl-2 are not protective of biological responsiveness. *Cancer Res* **54**: 1580–1586

Poeschla E and Wong-Staal F (1997) Antiviral and anticancer ribozymes. *Cur Opin Oncol* **6**: 601–606

Reid S, Cross R and Snow ED (1996) Combined hoechst 33342 and merocyanine 540 staining to examine murine B cell cycle sage, viability and apoptosis. *J Immunol Meth* **192**: 43–54

Rensing-Ehl A, Frei K, Flury R, Matiba B, Mariani SM, Weller M, Aebischer P, Krammer PH and Fontana A (1995) Local Fas/APO-1 (CD95) ligand-mediated tumor cell killing in vivo. *Eur J Immunol* **25**: 2253–2258

Saas P, Walker PR, Hahne M, Quiquerez AL, Schnuriger V, Perrin G, French L, Van Meir EG, de Tribolet N, Tschopp J and Dietrich PY (1997) Fas ligand expression by astrocytoma in vivo: maintaining immune privilege in the brain? *J Clin Invest* **99**: 1173–1178

Sato T, Irie S, Kitada S and Reed J (1995) FAP-1 a protein tyrosine phosphatase that associates with Fas. *Science* **268**: 411–415

Scanlon KJ, Jiao L, Funato T, Wang W, Tone T, Rossi JJ and Kashani-sabet M (1991) Ribozyme-mediated cleavage of c-fos mRNA reduces gene expression of DNA synthesis enzymes and metallothionein. *Proc Natl Acad Sci USA* **88**: 10591–10595

Seino KI, Kayagaki N, Tsukada N, Fukao K, Yagita H and Okumura K (1997) Transplantation of CD95 ligand-expressing grafts. Influence of transplantation site and difficulty in protecting allo- and xenografts. *Transplantation* **64**: 1050–1054

Shinohara H, Yagita H, Ikawa Y and Oyaizu N (2000) Fas drives cell cycle progression in glioma cells via extracellular signal-regulated kinase activation. *Cancer Res* **60**: 1766–1772

Shinoura N, Yoshida Y, Sadata A, Hanada KI, Yamamoto S, Kirino T, Asai A and Hamada H (1998) Apoptosis by retrovirus- and adenovirus-mediated gene transfer of Fas ligand to glioma cells: implications for gene therapy. *Hum Gene Ther* **9**: 1983–1993

Shiraki K, Tsuji N, Shiod T, Isselbacher KJ and Takahashi H (1997) Expression of Fas ligand in liver metastases of human colonic adenocarcinomas. *Proc Natl Acad Sci USA* **94**: 6420–6425

Strand S, Hofmann WJ, Hug H, Muller M, Otto G, Strand D, Mariani SM, Stremmel W, Krammer PH and Galle PR (1996) Lymphocyte apoptosis induced by CD95 (APO-1/Fas) ligand-expressing tumor cells – A mechanism of immune evasion? *Nature Med* **2**: 1361–1366

Suzuki I and Fink PJ (1998) Maximal proliferation of cytotoxic T lymphocytes requires reverse signaling through Fas ligand. *J Exp Med* **187**: 123–128

Takahashi T, Tanaka M, Inazawa J, Abe T, Suda T and Nagata S (1994) Human Fas ligand: gene structure, chromosomal location and species specificity. *Int Immunol* **6**: 1567–1574

Van Parijs L and Abbas AK (1996) Homeostasis and self-tolerance in the immune system: turning lymphocytes off. *Science* **280**: 243–248

Walker PR, Saas P and Dietrich PY (1998) Tumor expression of Fas ligand (CD95L) and the consequences. *Curr Opin Immunol* **10**: 564–572

Weller M, Frei K, Groscurth P, Krammer PH, Yonekawa Y and Fontana A (1994) Anti-Fas/APO-1 antibody-mediated apoptosis of cultured human glioma cells. Induction and modulation of sensitivity by cytokines. *J Clin Invest* **94**: 954–964

Yang BC and Yang TL (1998) Differential expression of cytokine genes and apoptosis in glioma cell lines upon exposure to bacteria and lipopolysaccharides. *J Microbiol Immunol Infect* **31**: 95–100

Yang BC, Wang YS, Wang CH, Lin HH, Tang MJ and Yang TL (1999) Insulin-elicited transient apoptosis in serum-starved glioma cells involved Fas/Fas-L and Bcl-2. *Cell Biol Intern* **23**: 533–540

Yang BC, Wang YS, HS Liu and Lin SJ (2000) Ras signaling is involved in the expression of Fas-L in glioma. *Lab Invest* **80**: 529–537

Zeytun A, Nagarkatti M and Nagarkatti PS (2000) Growth of FasL-bearing tumor cells in syngeneic murine host induces apoptosis and toxicity in Fas+ organs. *Blood* **95**: 2111–2117

 © 2001 Cancer Research Campaign
